# Can local application of vancomycin reduce surgical site infection rate after open lumbar fusion surgery?: A multicenter retrospective cohort study

**DOI:** 10.1097/MD.0000000000038664

**Published:** 2024-06-28

**Authors:** Zhendong Huan, Jijuan Zhao, Linkai Lei

**Affiliations:** a Department of Trauma Orthopedics, Yuhuangding Hospital Affiliated to Qingdao University, Yantai, Shandong Province, China; b Department of Laboratory, Yantai Hospital of Traditional Chinese Medicine, Yantai, Shandong Province, China; c Department of Spine Surgery, Yantaishan Hospital, Yantai, Shandong Province, China.

**Keywords:** local application, posterior open lumbar fusion, surgical site infection, vancomycin

## Abstract

Surgical site infection (SSI) after posterior open lumbar fusion (POLF) is a major concern for both surgeons and patients. We sought to explore whether local application of vancomycin could decrease the rate of SSI. We reviewed the clinical data of patients who underwent POLF between June 2015 and June 2022 at 3 spinal centers. Patients were divided into those who received local vancomycin (vancomycin group) and those who did not (non-vancomycin group). The SSI rates at 12 months postoperatively were compared between the 2 groups. Although a trend toward a lower infection rate was observed in the vancomycin group than in the non-vancomycin group; the difference was not statistically significant (3.6% vs 5.5%, *P* = .121). However, we found that the postoperative SSI rate was significantly lower in the vancomycin group than in the non-vancomycin group (4.9% vs 11.4%, *P* = .041) in patients ≥ 2 fused segments, while there was no significant difference in postoperative SSI rate in patients with single fusion segment (3.1% vs 3.6%, *P* = .706). The logistic regression analysis indicated that the SSI rate in the non-vancomycin group was approximately 2.498 times higher than that in the vancomycin group (*P* = .048, odds ratio: 2.498, 95% confidence interval: 1.011–6.617) in patients with ≥2 fused segments. In SSI patients with confirmed pathogens, the SSI rate of Gram-negative bacteria in the vancomycin group was significantly higher than that in the non-vancomycin group (10/14 [71.4%] vs 5/22 [31.8%]), whereas the SSI rate of Gram-positive bacteria in the vancomycin group was significantly lower than that in the non-vancomycin group (4/14 [28.6%] vs 15/22 [68.2%]). Local administration of vancomycin is recommended in patients with ≥2 fused segments as it may facilitate to reduce the postoperative rate of SSI after POLF. Additionally, the local use of vancomycin can decrease the Gram-positive bacterial infections but is not effective against Gram-negative infections, which indirectly leads to an increase in the proportion of Gram-negative infections in SSI patients with confirmed pathogens.

## 1. Introduction

The clinical application of lumbar fusion is increasing gradually around the world.^[1,2]^ The number of lumbar fusion patients in the United States has increased from 122,679 (60.4/100,000) in 2004 to 199,140 (79.8/100,000) in 2015. Additionally, over the past 12 years, the total hospital expenditure has increased by 177% to more than $10 billion in 2015, with an average of more than $50,000 per admission.^[3]^ In Finland, the rate of lumbar fusion increased from 9/100,000 person-years in 1997 to 30/100,000 person-years in 2018.^[4]^

Posterior open lumbar fusion (POLF) remains one of the mainstream surgeries for lumbar fusion.^[5]^ However, related surgical complications, particularly surgical site infections (SSI), plague many surgeons and patients.^[6]^ The probability of SSI occurring after lumbar fusion surgery ranges from 0.7% to 12%.^[7]^ Surgeons attempt to reduce postoperative infection rates by reducing the operative time (OT) and bleeding during surgery and strengthening aseptic procedures. In addition, the use of antibiotics such as vancomycin in the incision site has also been widely used to reduce the rate of SSI.^[8,9]^ Previous studies have found that local use of vancomycin powder can significantly reduce the rate of SSI after posterior lumbar surgery.^[8–18]^ However, recent studies have arrived at the opposite conclusion^[19–23]^ with few studies reporting that the local use of vancomycin not only reduces the postoperative rate of SSI but also leads to an increased infection rate of Gram-negative bacteria.^[20,21]^ In response to the controversy in previous studies, we sought to determine whether the local application of vancomycin during POLF could reduce the postoperative SSI rate. To avoid bias caused by a single center, this study included cases from 3 spine centers and analyzed the clinical outcomes of patients with and without the local use of vancomycin.

## 2. Materials and methods

We retrospectively analyzed the clinical data of patients who underwent POLF surgery at 3 spinal centers between June 2015 and June 2022. The patients were divided into vancomycin and non-vancomycin groups according to whether vancomycin was used locally during POLF surgery. This retrospective study was reviewed and approved by the hospital ethics committee. Owing to the retrospective nature of the study, patients were exempt from signing an informed consent form.

### 2.1. Inclusion criteria

Patients aged 18 years or older or 85 years or younger; those with POLF, which includes posterior lumbar interbody fusion (PLIF), transforaminal lumbar interbody fusion (TLIF), and posterolateral fusion (PLF) in patients with degenerative spinal disease or spondylolisthesis; those undergoing surgery with or without topical vancomycin use; patients with a follow-up period of at least 12 months.

### 2.2. Exclusion criteria

Patients aged < 18 years and > 85 years; those who underwent non-POLF surgery, which includes minimally invasive transforaminal lumbar interbody fusion (mis-TLIF), oblique lumbar interbody fusion (OLIF), anterior lumbar interbody fusion (ALIF), and lateral lumbar interbody fusion (LLIF); those with spinal fusion due to spinal trauma, infection, tuberculosis, or tumors; those with severe cardiovascular and cerebrovascular diseases or patients with preoperative weakness, such as anemia (female: hemoglobin concentration < 110 g/L, male: hemoglobin concentration < 120 g/L) and hypoproteinemia (the total protein content in plasma < 60 g/L, and plasma albumin < 30 g/L); those with autoimmune diseases such as ankylosing spondylitis and rheumatoid arthritis; those with immunodeficiency or long-term steroid or immunosuppressant use; those with poor wound healing compared to that with SSI. Poor wound healing and SSI are defined in detail below.

### 2.3. Patient information collection and recording

An independent, blinded clinical stuff collected patient’s demographics and clinical and surgical information. Demographics included sex, age, body mass index (BMI), comorbidities, smoking, alcohol consumption, and American Society of Anesthesiologists (ASA) score. Smoking was defined as a history of smoking before surgery and an average of more than 1 pack per week. Alcohol consumption was defined as drinking at least once per week on average. Diabetes was defined as a history of diabetes diagnosed before surgery. Additionally, surgical information, including surgical methods, OT, and patient fusion segments, was analyzed. Clinical information included the occurrence of postoperative SSI, type of infectious pathogen, and postoperative length of stay (LOS). LOS was defined as the time between surgery and discharge.

### 2.4. Definition of SSI

SSI were diagnosed according to the guidelines established by the Centers for Disease Control and Prevention.^[24]^ Whether a patient has experienced an SSI can be determined from the disease course and surgical records. Patients who experienced SSI within 12 months after surgery were included in this study. SSI are characterized by local swelling, pain, and persistent secretion at the surgical site. When screening cases, it is necessary to distinguish between patients with SSI and those with poor wound healing. Postoperative SSI was defined as the development of pathogens in wound secretions and incisions. In addition, patients who underwent internal debridement of the incision but did not present with pathogens on the surface or inside the incision and sustained poor healing of the incision with significant exudation were also classified as having SSI. Patients who do not cultivate pathogens on the surface of the incision but only have delayed wound healing (delayed healing refers to the patient’s wound healing time being longer than the general wound healing time; however, ultimately the incision is healed) and those who have not undergone internal debridement of the incision are considered to have poor wound healing, rather than SSI. Patients who only underwent wound surface debridement were not considered to have SSI because some patients may need to suture the skin again due to poor healing of the incision. Therefore, only patients who required internal debridement of the incision with or without pathogenic evidence were considered to have potential SSI.

### 2.5. Surgical procedure

Surgical procedures include the 3 most common posterior lumbar fusion procedures: PLIF, TLIF, and PLF. All surgical techniques were performed according to the procedures reported in the literature.^[5]^ All patients were administered intravenous antibiotics 30 minutes before surgery. Patients with an operation lasting > 3 hours received an additional set of intravenous antibiotics. All procedures were performed under general anesthesia by the experienced surgeon. All the patients underwent decompression, bone graft fusion, and instrumental fixation surgery.

### 2.6. Local application of vancomycin

In the vancomycin group, 2 grams of vancomycin powder was sprinkled evenly on the surgical site after the operation and the incision was sutured layer-by-layer. The incision was sutured directly after surgery in patients who did not receive vancomycin.

### 2.7. Statistical methods

For continuous variables such as age, BMI, OT, fusion segment, and LOS, the Student *t* (normally distributed variables, mean ± standard deviation) or Wilcoxon rank sum test (non-normally distributed variables, Median (interquartile range)) were used to compare the differences between the 2 groups. For categorical variables such as sex, ASA grade, smoking, drinking, comorbidities, surgical methods, infection rate, the chi-square or Fisher-exact tests were used to compare the differences between the 2 groups. Statistical significance was set at *P* < .05. Statistical analyses were performed using SPSS Statistics.22 software (IBM Corp., Armonk, NY).

## 3. Results

**Figure 1.**
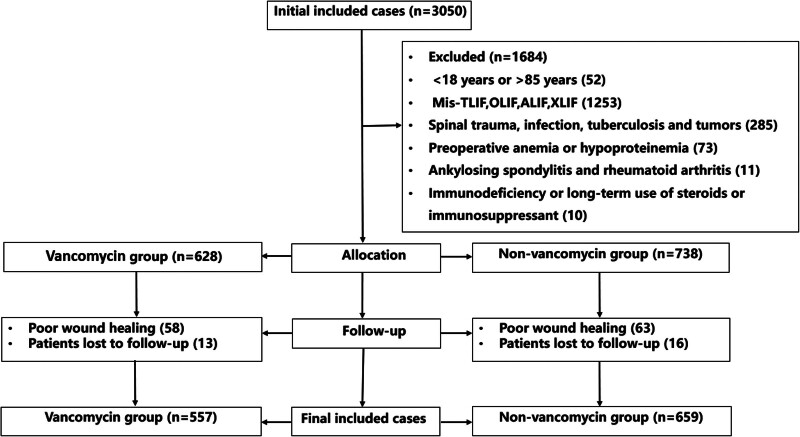
The procedure for patient exclusion and inclusion.

The procedure for participant selection is illustrated in Figure 1. Through a preliminary screening of the hospital case system, 3050 patients were enrolled in this study. After screening using the inclusion and exclusion criteria, 52 patients aged < 18 years or > 85 years; 1253 cases of mis-TLIF, OLIF, ALIF, or XLIF; 285 patients with spinal trauma, infection, tuberculosis, or tumors; 73 cases of preoperative anemia or hypoproteinemia; 11 cases of ankylosing spondylitis and rheumatoid arthritis; and 10 cases of immunodeficiency or long-term use of steroids or immunosuppressants were excluded. A total of 121 patients with poor wound healing and 29 patients lost to follow-up were excluded from the study. Ultimately, 1216 patients were included in this study. Among them, there were 557 patients in the vancomycin group and 659 patients in the non-vancomycin group. No significant differences were noted in the sex, age, BMI, smoking, drinking, or comorbidities (diabetes or hypertension) between the 2 groups. In addition, there were no significant differences in the ASA grade, diabetes, hypertension, surgical methods, fusion segment, OT, or LOS between the 2 groups (Table 1).

**Table 1:** Patients’ clinical parameters of the 2 groups.

Subgroup	Vancomycin group (N = 557)	Non-vancomycin group (N = 659)	*P* value
Sex			
Men	287 (51.5%)	330 (50.1%)	.614
Women	270 (48.5%)	329 (49.9%)	
Age (yr)	57.26 ± 10.45	56.73 ± 10.54	.378
BMI (kg/m^2^)	24.98 ± 3.03	25.11 ± 3.07	.464
ASA score			
1	52 (9.3%)	73 (11.1%)	.608
2	438 (78.6%)	509 (77.2%)	
3	67 (12.0%)	71 (11.7%)	
Fusion segment	1.36 ± 0.76	1.39 ± 0.79	.528
Operative time	162.90 ± 28.14	161.66 ± 29.85	.459
Surgical methods			
PLIF	288 (51.7%)	322 (48.9%)	.227
TLIF	244 (43.8%)	316 (48.0%)	
PLF	25 (4.5%)	21 (3.2%)	
Smoking	75 (13.5%)	95 (14.4%)	.634
Consumption of alcohol	96 (17.2%)	104 (15.8%)	.496
Diabetes	72 (12.9%)	68 (8.5%)	.156
Hypertension	158 (24.4%)	161 (28.4%)	.120
Length of hospital	5.47 ± 1.74	5.55 ± 2.16	.484

ASA = American Society of Anesthesiologists, BMI = body mass index, PLF = posterolateral fusion, PLIF = posterior lumbar interbody fusion, TLIF = transforaminal lumbar interbody fusion.

The infection rates in the 2 groups are shown in Table 2. Overall, 3.6% (20/557) patients were occurred in the vancomycin group and 5.5% (36/659) patients were occurred in the non-vancomycin group. A trend toward a lower infection rate was observed in the vancomycin group than in the non-vancomycin group; however, the difference was not statistically significant (3.6% vs 5.5%, *P* = .121). We found that the postoperative infection rate was significantly lower in vancomycin group than in non-vancomycin group (vancomycin group: 4.9% vs non-vancomycin group: 11.4%, *P* = .041) in patients with ≥2 fused segments. No significant difference was noted in the postoperative infection rate between the 2 groups of patients with one fusion segment (vancomycin group: 3.1% vs vancomycin group: 3.6%, *P* = .706). The logistic regression analysis indicated that the infection rate in non-vancomycin group was approximately 2.498 times higher than that in the vancomycin group (*P* = .048, odds ratio (OR) = 2.498, 95% confidence interval (CI): 1.011–6.617).

**Table 2:** The infection rates of the 2 groups.

Subgroup	Infection (yes)	Infection (no)	*P* value
All patients			
Vancomycin group (N = 557)	20 (3.6%)	537 (96.4%)	.121
Non-vancomycin group (N = 659)	36 (5.5%)	623 (94.5%)	
Patients (single fusion segment)			
Vancomycin group (N = 414)	13 (3.1%)	401 (96.9%)	.706
Non-vancomycin group (N = 501)	18 (3.6%)	483 (96.4%)	
Patients (≥2 fusion segments)			
Vancomycin group (N = 143)	7 (4.9%)	136 (95.1%)	.041
Non-vancomycin group (N = 158)	18 (11.4%)	140 (88.6%)	

The types of pathogens associated with SSI in the 2 patient groups are shown in Table 3. The most common SSI pathogens in the vancomycin and non-vancomycin groups were Pseudomonas aeruginosa (14.3%, 2/14) and Staphylococcus aureus (13.6%, 3/22). No significant difference was noted in the overall rate of Gram-negative infections between the 2 groups (vancomycin group: 10/557 [1.8%] vs non-vancomycin group: 7/659 [1.1%], *P* = .278); however, the overall rate of Gram-positive infections in the vancomycin group was significantly lower than that in the non-vancomycin group (vancomycin group: 4/557 [0.7%] vs non-vancomycin group: 15/659 [2.3%], *P* = .036).

**Table 3:** Patients’ infection pathogens of the 2 groups.

Subgroup	Vancomycin group (N = 20)	Non-vancomycin group (N = 36)	*P* value
*Staphylococcus aureus*	1	3	
*Staphylococcus epidermidis*	1	2	
*Staphylococcus capitis*		1	
*Pseudomonas aeruginosa*	2	2	
*Human staphylococcus*	1	1	
*Streptococcus viridans*		1	
*Streptococcus constellatus*	1	1	
*Staphylococcus coriolis urealyticus*		1	
*Enterobacter cloacae*	1	1	
*Streptococcus suis*		1	
*Anaerobic streptococcus*		1	
*Morganella morgana*	1		
*Escherichia coli*	1	2	
*Proteus mirabilis*	1	1	
*Citrobacter freundii*	1		
*Enterococcus faecalis*		1	
*Enterococcus faecium*		2	
*Acinetobacter baumannii*	1		
*Bacillus cereus*	1		
*Escherichia faecalis*	1	1	
Pathogen negative	6	13	
Gram-negative bacteria	10 (1.8%)	7 (1.1%)	.278
Gram-positive bacteria	4 (0.7%)	15 (2.3%)	.036

No specific pathogen was detected in 30% (6/20) of the SSI patients in the vancomycin group and 36% (13/36) in the non-vancomycin group. In SSI patients with confirmed pathogens (totally 36 cases), the infection rate of Gram-negative bacteria in the vancomycin group was significantly higher than that in the non-vancomycin group (10/14 [71.4%] vs 5/22 [31.8%]). The infection rate of Gram-positive bacteria in the vancomycin group was significantly lower than that in the non-vancomycin group (4/14 [28.6%] vs 15/22 [68.2%]) (Table 4).

**Table 4:** Types of bacterial infections in SSI patients with confirmed pathogens.

Subgroup	Vancomycin group (N = 14)	Non-vancomycin group (N = 22)	*P* value
Gram-negative bacteria	10 (71.4)	7 (31.8%)	.039
Gram-positive bacteria	4 (28.6%)	15 (68.2%)	

## 4. Discussion

Local use of vancomycin in incisions for lumbar fusion remains controversial.^[8–23]^ A recent one-to-one propensity score-matched analysis (444 cases) by Ushirozako et al^[13]^ found that the local use of vancomycin powder was useful in reducing the risk of SSI after posterior spinal surgery by half (2.7% vs 5.4%, *P* = .041, OR = 0.486, 95% CI: 0.243–0.972), without adverse events, which is a safe and effective procedure for SSI prevention. However, a recent randomized prospective study of 375 cases (187 cases: local use of vancomycin, 188 cases: no use of vancomycin) by Salimi et al^[21]^ found that the local use of vancomycin did not reduce the risk of SSI and can potentially increase the rate of Gram-negative bacterial infections. However, their study included both instrumented and noninstrumented cases. In our experience, the SSI rate tends to be higher in cases of instrumented fusion. In this study, we investigated whether the local use of vancomycin in wounds reduces the rate of SSI after POLF. We found no significant differences between the vancomycin-treated and vancomycin-free groups in single-segment POLF. However, in the patients with ≥2 segments, the local use of vancomycin can significantly reduce the rate of SSI, which will provide an important reference for clinical treatment. This may not be difficult to explain, as compared to single-segment fusion surgery, lumbar fusion with 2 or more segments means a longer OT, more intraoperative blood loss, and greater surgical trauma, as well as a potentially higher rate of SSI.

Vancomycin powder is most commonly spread evenly across surgical sites. However, this method may have some drawbacks, such as difficulty in evenly distributing the powder, resulting in dead spots. This may also be one of the reasons why some studies have found that the local use of vancomycin does not significantly reduce SSI. Higashi et al recently developed a new technology (local administration of vancomycin suspended in fibrin glue) to achieve a better distribution and release of vancomycin,^[11]^ which was significantly associated with lower SSI rates in patients who underwent spinal instrumentation surgery. The current method of administering vancomycin powder may not be the best choice and new scientific methods of administration should be developed to achieve optimal clinical effects. In addition, the potential complications of local vancomycin use, such as transient hearing loss resulting from ototoxicity, nephropathy, and supratherapeutic vancomycin exposure resulting from systemic absorption, should also be mentioned to the patient, although related complications are rare.^[25]^

Notably, the use of vancomycin results in additional costs. However, the extra cost is minimal compared with the extra cost due to the prolonged or severe SSI. Godil et al^[26]^ found that the local use of vancomycin can significantly reduce the rate of SSI after spinal fusion (0% vs 13%, *P* = .02). Additionally, the local use of vancomycin powder can lead to cost savings of $438,165 per 100 posterior spinal fusions. Emohare et al^[27]^ found that among 96 patients treated with vancomycin locally, no patient underwent re-surgery for SSI. Of the 207 patients who did not receive vancomycin locally, a total of 7 patients underwent additional surgery for SSI. In addition, patients who received local vancomycin only required a total additional cost of $1152 (96 × 12), while 3% (7/207) of patients who did not receive local vancomycin spent an additional $573,897 due to SSI. Although we did not calculate the average and total costs of the 2 groups, these costs may have been affected by the surgical segment, type and price of internal fixation, and type of insurance. There is reason to believe that the local use of vancomycin is a cost-effective measure for SSI prevention.

The main effect of vancomycin is the inhibition of the proliferation of Gram-positive bacteria. A study by Adogwa et al^[28]^ showed that the local use of vancomycin can reduce the rate of infection with Gram-positive bacteria; however, it increases the SSI rate of infection with Gram-negative bacteria. However, Chotai et al^[29]^ found that the incidence of SSIs due to *Staphylococcus aureus* was significantly reduced in patients who received intraoperative vancomycin for spinal surgery. In addition, no vancomycin-resistant pathogens were identified in the patients who received intraoperative vancomycin powder and subsequently developed *S aureus* SSI. However, Gram-negative SSI (28% vs 7%) and culture-negative effusion (16% vs 5%) were more common in patients treated with vancomycin. In addition, Salimi et al^[21]^ found that the local use of vancomycin cannot reduce the proportion of Gram-positive SSI but destroys the microbial balance in the incision, leading to an increase in the rate of Gram-negative infection. In this study, we found a significantly lower overall rate of Gram-positive bacterial infections in the vancomycin group than that in the non-vancomycin group (4/557 [0.7%] vs 15/659 [2.3%], *P* = .036). Although a slightly higher overall rate of Gram-negative infections was observed in the vancomycin group, the difference was not significant (10/557 [1.8%] vs 7/659 [1.1%], *P* = .278). Additionally, in SSI patients with confirmed pathogens (totally 36 cases), the infection rate of Gram-negative bacteria in the vancomycin group was significantly higher than that in the non-vancomycin group (10/14 [71.4%] vs 5/22 [31.8%]). The infection rate of Gram-positive bacteria in the vancomycin group was significantly lower than that in the non-vancomycin group (4/14 [28.6%] vs 15/22 [68.2%]). This indicates a significant inhibitory effect of vancomycin on Gram-positive bacteria that leads to an increase in the proportion of Gram-negative bacterial infections. Therefore, in addition to vancomycin, it may be necessary in the future to add an additional antibiotic to the incision, mainly against Gram-negative bacteria, to further reduce the rate of Gram-negative infection.

A recent retrospective study by Ying et al^[30]^ found that bacterial cultures were positive in only 47 (42.3%) of 111 patients with SSI after lumbar spinal surgery. In this study, except for SSI patients in whom pathogens were detected, no specific pathogen was detected in 30% (6/20) of the patients in the vancomycin group and 36% (13/36) in the non-vancomycin group. A common feature is that the surgical incision is accompanied by swelling, pain, and persistent secretions, and the incision cannot heal after dressing changes. Subsequently, all patients underwent debridement. However, no pathogens were detected after culturing the tissue extracted from the incision, probably because the local use of vancomycin, to some extent, inhibited bacterial growth at the surgical site. In addition, before debridement surgery, patients are usually treated empirically with intravenous antibiotics, which can cause bacterial infections and result in negative tissue culture results.

## 5. Limitations

Although this was a multicenter study, its retrospective nature led to inevitable bias in the selection of cases. The patients were not randomly assigned to use or not to use vancomycin locally. Instead, the decision to use vancomycin locally was based on the surgeon’s cognition and experience, the habits of different physicians in managing patients, and their preoperative expectancy for the occurrence of postoperative SSI in patients. Additionally, although there are few prospective studies on the local use of vancomycin, the sample sizes in these studies were generally small. Larger sample sizes are required to further define the role of local vancomycin administration in reducing the probability of SSI. In the future, multicenter, prospective, large-sample studies are needed to further define the role of topical vancomycin in reducing SSI.

## 6. Conclusion

Local administration of vancomycin is recommended in patients with ≥2 fused segments as it may help in reducing the postoperative rate of SSI after POLF. Additionally, the local use of vancomycin can decrease the Gram-positive bacterial infections but is not effective against Gram-negative infections, which indirectly leads to an increase in the proportion of Gram-negative infections in SSI patients with confirmed pathogens.

## Author contributions

**Data curation:** Zhendong Huan, Jijuan Zhao, Linkai Lei.

**Investigation:** Zhendong Huan, Jijuan Zhao.

**Writing – original draft:** Zhendong Huan.

**Methodology:** Jijuan Zhao, Linkai Lei.

**Software:** Jijuan Zhao.

**Writing – review & editing:** Jijuan Zhao, Linkai Lei.

**Conceptualization:** Linkai Lei.

**Supervision:** Linkai Lei.
